# The Exocyst Protein Sec10 Interacts with Polycystin-2 and Knockdown Causes PKD-Phenotypes

**DOI:** 10.1371/journal.pgen.1001361

**Published:** 2011-04-07

**Authors:** Ben Fogelgren, Shin-Yi Lin, Xiaofeng Zuo, Kimberly M. Jaffe, Kwon Moo Park, Ryan J. Reichert, P. Darwin Bell, Rebecca D. Burdine, Joshua H. Lipschutz

**Affiliations:** 1 Department of Medicine, University of Pennsylvania, Philadelphia, Pennsylvania, United States of America; 2 Department of Molecular Biology, Princeton University, Princeton, New Jersey, United States of America; 3 Department of Anatomy and BK 21 Project, Kyungpook National University, Daegu, Republic of Korea; 4 Department of Medicine, Medical University of South Carolina, Charleston, South Carolina, United States of America; 5 Department of Medicine, Philadelphia Veterans Affairs Medical Center, Philadelphia, Pennsylvania, United States of America; Washington University School of Medicine, United States of America

## Abstract

Autosomal dominant polycystic kidney disease (ADPKD) is characterized by formation of renal cysts that destroy the kidney. Mutations in PKD1 and PKD2, encoding polycystins-1 and -2, cause ADPKD. Polycystins are thought to function in primary cilia, but it is not well understood how these and other proteins are targeted to cilia. Here, we provide the first genetic and biochemical link between polycystins and the exocyst, a highly-conserved eight-protein membrane trafficking complex. We show that knockdown of exocyst component Sec10 yields cellular phenotypes associated with ADPKD, including loss of flow-generated calcium increases, hyperproliferation, and abnormal activation of MAPK. Sec10 knockdown in zebrafish phenocopies many aspects of polycystin-2 knockdown—including curly tail up, left-right patterning defects, glomerular expansion, and MAPK activation—suggesting that the exocyst is required for *pkd2* function *in vivo*. We observe a synergistic genetic interaction between zebrafish *sec10* and *pkd2* for many of these cilia-related phenotypes. Importantly, we demonstrate a biochemical interaction between Sec10 and the ciliary proteins polycystin-2, IFT88, and IFT20 and co-localization of the exocyst and polycystin-2 at the primary cilium. Our work supports a model in which the exocyst is required for the ciliary localization of polycystin-2, thus allowing for polycystin-2 function in cellular processes.

## Author Summary

ADPKD, the most common potentially lethal monogenetic disorder, is caused by mutations in PKD1 and PKD2. We are beginning to appreciate the important roles these gene products, and others, play in cilia, which are thin rod-like organelles projecting from the cell surface. Defects in cilia function are associated with a variety of human diseases, including all variants of polycystic kidney disease. Despite intense study of cilia and how they influence disease, it is not understood how proteins are targeted and delivered to cilia. Our work provides the first link between the exocyst, a conserved eight-protein complex involved in protein localization, and a disease gene, PKD2. Knockdown of the exocyst protein Sec10 results in a number of cellular- and cilia-related phenotypes that are also seen upon *pkd2* loss—both in kidney cells and zebrafish. We then demonstrate specific genetic and biochemical interactions between *sec10* and *pkd2*. We further show that Sec10 interacts with other ciliary proteins, such as IFT20 and IFT88. From this work, we propose that the exocyst is involved in bringing multiple types of ciliary proteins to the cilium. Given that the exocyst is required for cilia structure and function, the exocyst may play a role in cilia-related human diseases.

## Introduction

ADPKD is the most common potentially lethal monogenetic disorder, affecting 12 million people worldwide [1]. ADPKD is characterized by the development of numerous renal cysts, which greatly increase kidney size, perturb kidney function, and eventually lead to kidney failure. While we know that mutations in PKD1 and PKD2 cause ADPKD [2], [3], we are only beginning to understand how the proteins—polycystin-1 and polycystin-2—regulate the cellular phenotypes associated with cystogenesis.

Interactions between polycystin-2, a calcium-permeable cation channel [4], [5], and polycystin-1 may act to regulate calcium signaling in normal kidney cells [6]. Consistent with calcium regulation being relevant to cystogenesis, ADPKD cells show a lower basal intracellular calcium concentration [7]. Furthermore, altered calcium regulation has been linked, through cyclic AMP (cAMP) signaling, to phenotypes observed during cystogenesis, such as increased cell proliferation and abnormal fluid secretion. Addition of cAMP agonists cause ADPKD cells, but not normal kidney cells, to stimulate proliferation via the MAPK pathway [6], [8], [9].

Growing evidence suggests that the cilium is an important site of polycystin function. Kidney tubular epithelial cells have a single non-motile primary cilium that acts as a mechanosensor, triggering a rise in intracellular calcium in response to fluid flow [10], [11]. Polycystins-1 and -2 localize to the primary cilium of kidney cells [12], [13], and the calcium response to fluid flow requires polycystin function [14]. Consistent with the idea that mechanosensation is relevant to cystogenesis, ADPKD cells are unresponsive to fluid flow [15].

Research in animal models suggests that cilia play important roles, not only during adult kidney function, but also throughout early embryonic development (reviewed in [16]). Zebrafish has been increasingly used as a model organism to expand our understanding of the *in vivo* function of ciliary proteins through studies utilizing mutants that affect cilia, and morpholino antisense knockdown of ciliary proteins. Loss of intraflagellar transport proteins (reviewed in [17]), which are required for cilia assembly, results in body axis curvatures (“curly tails”), left-right defects, pronephric cysts, edema, and small eye phenotypes [18]-[21]. Other mutants that show disrupted cilia length or motility similarly show curly tails, left-right defects, and pronephric cysts [20], [22]-[26].

These phenotypes, which comprise the range of cilia-related phenotypes in zebrafish, suggest that proper cilia formation and/or function is required for multiple developmental processes. The mechanistic relationship connecting cilia to each phenotype is understood to differing degrees depending on the specific phenotype. The connection is well understood for left-right patterning and pronephric development. Left-right patterning governs the stereotypical positioning of organs, which is preceded and directed by left-sided expression of the Nodal signaling pathway (reviewed in [27]). The asymmetric expression of the *nodal* genes *spaw*, *lefty1*, and *lefty2* in zebrafish is itself thought to be established by cilia-dependent fluid flow in Kupffer's vesicle [18], [28]. Indeed, mutants that show disrupted cilia length or flow in Kupffer's vesicle subsequently show randomized *nodal* gene expression and left-right defects [18], [28]. Cilia in the pronephric tubules are similarly thought to be important for pronephric development such that perturbations in motility result in tubule dilations and cystogenesis [25], [26].

Research into *pkd2* function in zebrafish has further strengthened the idea that polycystin-2 functions in the cilium. Knockdown of *pkd2* by morpholino [29]-[31] or in mutants [20], [31] produces phenotypes that are consistent with a role in cilia function: curly tails, left-right defects, pronephric cysts, and edema. Indeed, polycystin-2 is expressed in Kupffer's vesicle, and mutations in *pkd2* lead to defects in left-right patterning in zebrafish and mice [29]-[32]. However, *pkd2* is unique in zebrafish for a number of reasons. First, it is the only reported mutant to consistently display a curly tail up phenotype [20], [29]-[31], as opposed to the typical curly tail down phenotype of other cilia mutants. Secondly, *pkd2* knockdown does not produce observable defects in cilia structure [29]-[31] or motility [25], [30]. Therefore, *pkd2* is likely to be important for cilia function in a way that is distinct from a role in cilia formation, maintenance, or motility. For example, it has been proposed that *pkd2* may play a specific mechanosensory role related to calcium regulation during left-right patterning in mice [33].

While we are beginning to identify the roles ciliary proteins play in diverse biological processes, there is little known about how these proteins are transported to the cilium [34]. The exocyst, originally identified in *S. cerevisiae* [35], is a highly conserved 750kD eight-protein complex known for the targeting and docking of vesicles carrying membrane proteins [36]. It is comprised of Sec3, Sec5, Sec6, Sec8, Sec10, Sec15, Exo70, and Exo84 [37]. Notably, in addition to being found near the tight junction, we localized exocyst proteins to the primary cilium in kidney cells [38], [39]. Sec10 and Sec15 are the most vesicle-proximal of the exocyst components. Sec10 has been shown to directly bind to Sec15, which, in turn, directly binds Sec4, a Rab GTPase on the surface of transport vesicles. Sec10 then acts as a “linker”, by binding the other exocyst components through Sec5 [40]. Our previous studies suggested that the exocyst would no longer be able to bind Sec15 and target/dock transport vesicles without Sec10, and would, instead, disintegrate and be degraded. Importantly, we showed that knockdown of exocyst Sec10 in Madin-Darby canine kidney (MDCK) cells abrogated ciliogenesis, while Sec10 overexpression enhanced ciliogenesis. Furthermore, Sec10 knockdown caused abnormal cystogenesis when the cells were grown in a collagen matrix, and decreased the levels of other exocyst components and the intraflagellar transport protein 88 (IFT88). This was in contrast to knockdown of exocyst components Sec8 and Exo70, which had no effect on ciliogenesis, cystogenesis, or levels of other exocyst components [39]. These data uncovered a role for the exocyst, and especially the Sec10 component, in building the primary cilium. Given its known role in trafficking proteins to the plasma membrane [41]-[44], we have proposed that Sec10 and the exocyst may be required in the cilium to target and dock vesicles carrying proteins important for ciliogenesis.

Here we show that Sec10 knockdown, *in vitro* in MDCK cells and *in vivo* in zebrafish, results in phenotypes associated with loss of polycystins and ADPKD. We specifically demonstrate a genetic and biochemical interaction between Sec10 and polycystin-2, as well as show co-localization at the primary cilium, providing further evidence that the exocyst is important for polycystin-2 function. Furthermore, we show biochemical interactions between Sec10 and the ciliogenesis proteins IFT88 and IFT20. Our results demonstrate that the exocyst is required for *pkd2* function in the cell. Together with our previous results, these data suggest that the exocyst is important for maintaining both cilia structure and function. Exocyst dysfunction may therefore contribute to ciliopathies including ADPKD, and Sec10 may represent a novel target for the development of effective treatments.

## Results

### Exocyst Sec10 knockdown leads to a cellular phenotype similar to ADPKD cells

**Figure 1:**
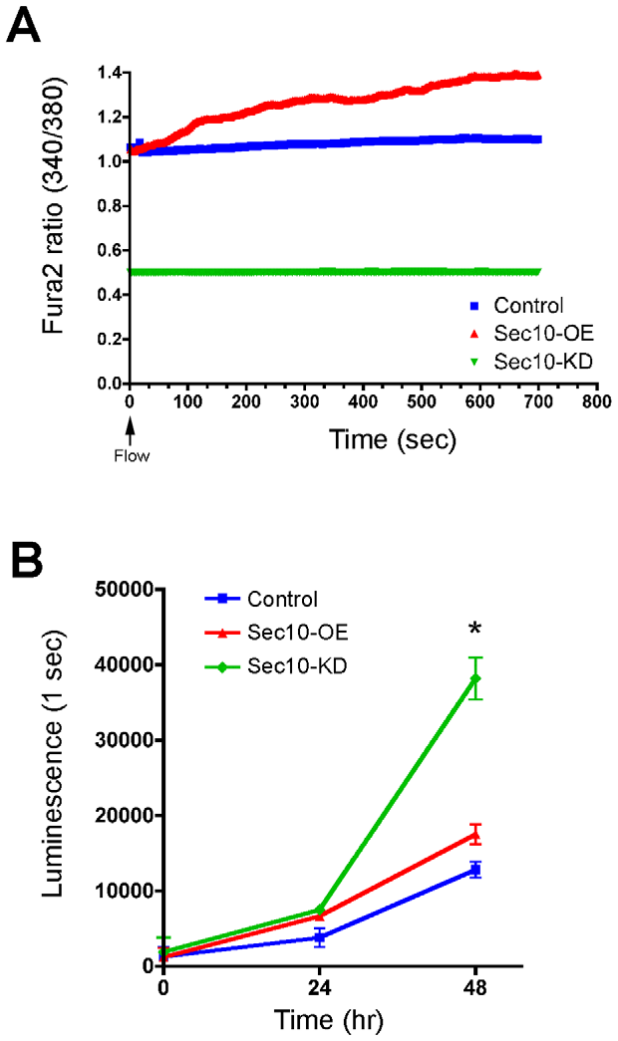
Sec10 knockdown in MDCK cells leads to ADPKD-like phenotypes. A) Intracellular calcium levels were plotted as the ratio of Fura-2 fluorescence 340/380 nm, as a function of time in seconds. Confluent cells were exposed to a constant shear fluid flow rate of 5 ml/minute beginning at time = 0 seconds (arrow). Intracellular calcium and response to flow were decreased in Sec10 knockdown cells (green) and increased in Sec10-overexpressing cells (red), compared to wild-type MDCK cells (blue). B) Cell proliferation at 24 and 48 hours was plotted based on a cell titer luminescence assay, as a function of time, and was increased in Sec10 knockdown cells (* = p<0.01).

Given that loss of polycystin-2 leads to ADPKD, we first determined whether exocyst Sec10 knockdown or overexpression in MDCK cells produced ADPKD-like phenotypes. Primary cultures of ADPKD cells fail to show the expected rise in calcium levels in response to a shear flow [7], [15]. After growing these MDCK cell lines to confluent monolayers on Transwell filters, conditions that we have previously shown results in ciliation [39], we measured steady state levels of intracellular calcium using the Fura-2 indicator. We then tested whether cells exposed to a constant 5 ml/minute flow rate over the apical surface responded appropriately with an increase in calcium levels. Sec10 knockdown cells showed a significantly lower basal calcium level than both control MDCK and Sec10-overexpressing cells, and calcium levels in the Sec10 knockdown cells also failed to increase in response to fluid flow (0.2% increase in Sec10 knockdown versus 5.8% in control and 26.2% in Sec10-overexpressing cells) (Figure 1A). Thus, Sec10 knockdown cells do indeed produce phenotypes similar to that observed in ADPKD cells [7], [15]. The reason for the limited increase in calcium in response to fluid flow in control T23 MDCK cells, that constitutively express the tetracycline transactivator that drives the Sec10 shRNA, is likely due to the fact that ciliogenesis, for unknown reasons, is sporadic—with the literature describing from ∼30% of T23 MDCK cells being ciliated (as we see [39]), to as few as 14.6% being ciliated [45]. The loss of mechanosensation in Sec10 knockdown cells is consistent with the loss of cilia in these cells [39], [46]. Similarly, since Sec10-overexpressing cells display longer cilia [39], the increased calcium response may reflect a heightened mechanosensory capability of those cilia.

Cellular hyperproliferation is another major feature of ADPKD cells [47], so we investigated whether the abnormal calcium level observed in Sec10 knockdown cells was associated with hyperproliferation. Using a luminescence-based viability assay, Sec10 knockdown cells showed an increased rate of proliferation after 48 hours (Figure 1B). Sec10-overexpressing cells, by contrast, showed a relatively normal rate of proliferation.

### Exocyst Sec10 knockdown leads to activation of MAPK

**Figure 2:**
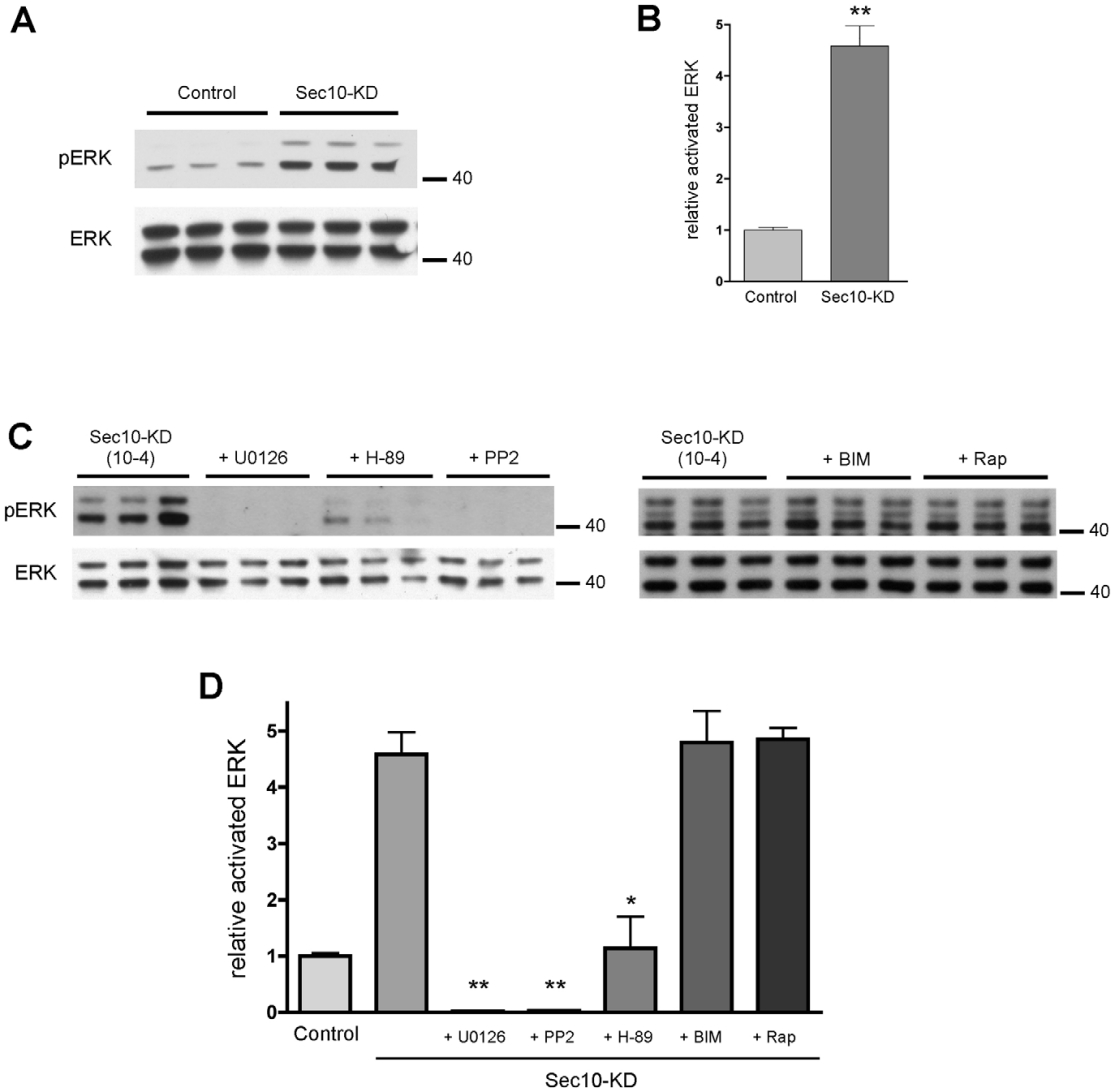
Sec10 knockdown in MDCK cells leads to activation of MAPK. (A) Increased active, or phosphorylated, ERK (pERK) levels are seen by Western blot following Sec10 knockdown (Sec10-KD) in MDCK cells. (B) Quantification of results in (A) (** p<0.001). (C) Increased pERK in Sec10-KD cells is completely blocked by addition of the MEK inhibitor U0126 and the src inhibitor PP2, while the cAMP-activated PKA inhibitor H-89 reduces pERK levels to approximately normal levels. Other kinase inhibitors, including the PKC inhibitor bisindolylmaleimide I (BIM), and the mTOR inhibitor Rapamycin (RAP), showed no effect on pERK levels in these cells. (D) Quantification of results in (C) (* p<0.01, ** p<0.001). Experiments were run in triplicate, and equal amounts of protein, as determined by BCA, were loaded in each lane. Antibodies against pERK and total ERK (ERK) were used. The densities of the pERK bands were normalized to the densities of the corresponding total ERK bands, and the ratio of pERK:ERK was normalized to control MDCK cells.

The mitogen activated protein kinase (MAPK) pathway is activated during cell proliferation and kidney development [48], [49]. It has been shown in some mouse models of ADPKD that the MAPK pathway is activated, and that blockage of extracellular-signal regulated kinase (ERK) slows the development of polycystic kidney disease [50]. It has been theorized that the decreased intracellular calcium in primary ADPKD cells, resulting from the dysfunctional primary cilia, leads to increased cyclic AMP (cAMP) and, therefore, protein kinase A (PKA) activity, with downstream MAPK pathway hyperactivation [51]-[53]. We therefore tested whether the low intracellular calcium and hyperproliferative phenotypes of Sec10 knockdown cells were accompanied by activation of the MAPK pathway. The MAPK pathway involves the phosphorylation cascade of Raf, MEK, and ERK. By Western blot analysis, phosphorylated ERK (pERK)—a measure of MAPK activation—was increased in Sec10 knockdown cells relative to normal cells by 4.6-fold (Figure 2A and 2B). This increase in pERK was blocked completely with a 1-hour treatment of a MEK inhibitor (U0126) and a src-family inhibitor (PP2). Treatment with a cAMP-activated PKA inhibitor (H-89) partially blocked activated ERK, restoring levels of pERK to approximately that of control MDCK cells. Treatment with other kinase inhibitors, including a PKC inhibitor (BIM) and an mTOR inhibitor (Rapamycin), were ineffective in blocking overactivation of pERK in Sec10 knockdown cells (Figure 2C and 2D), suggesting that the pERK increase was specific for the MAPK pathway. These data support the idea that the increased pERK observed in Sec10 knockdown cells is due to the combined upstream activities of PKA, Src, and MEK.

Together, our *in vitro* results show that Sec10 knockdown cells display many cellular phenotypes shared with ADPKD cells: from abnormal calcium regulation associated with an insensitivity to fluid flow, to increased proliferation associated with MAPK activity.

### Exocyst Sec10 is required for normal pronephric development in zebrafish

To determine how *sec10* affects cilia and cilia-related processes *in vivo*, we utilized morpholinos (MOs) to knockdown zebrafish Sec10 (zfSec10) levels. Our first start-site morpholino was ineffective at knocking down zfSec10 levels (see Materials and Methods). Since exocyst Sec8 knockout mice display very early embryonic lethality, well before kidney development occurs [54], we did not focus on developing working start-site morpholinos because these would affect both maternal and zygotic transcripts and could cause early phenotypes that would preclude any analysis of specific phenotypes. Thus, we utilized splice-site morpholinos, which would bypass any early general requirement for the exocyst and allow us to focus on later tissue-specific effects of Sec10 knockdown.

**Figure 3:**
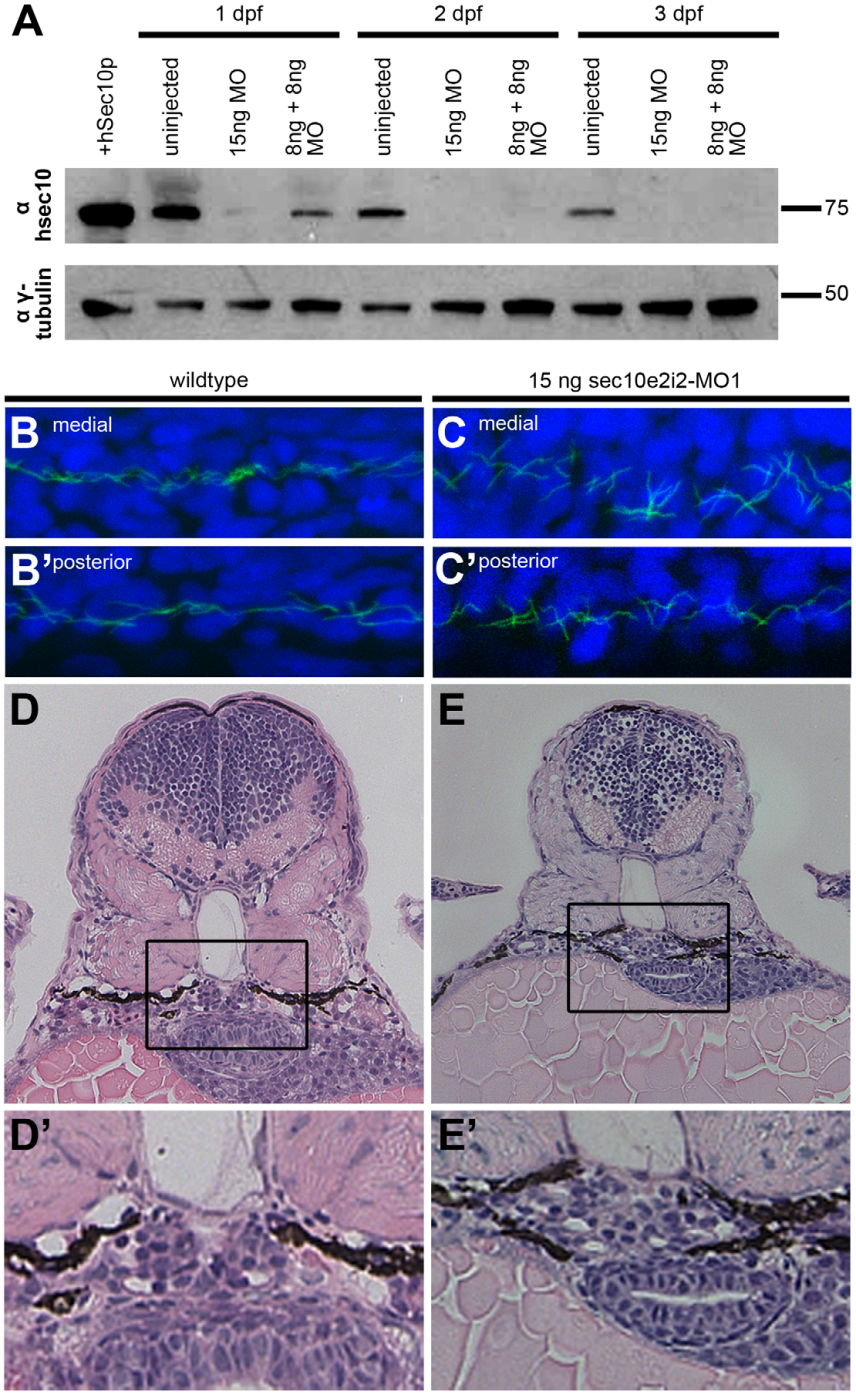
sec10MO embryos show abnormal pronephric development. (A) Immunoblots showing the 1-3 dpf time course of zfSec10 knockdown in sec10MO embryos. sec10MO injections can lead to abnormal-appearing embryos (Table 1), but in this blot, 3 dpf sec10MO embryo lysates were isolated only from embryos without any obvious morphological defects. This demonstrates that these embryos still have strong knockdown of the zfSec10 protein. Lysates from abnormal-appearing embryos showed similar levels of zfSec10 knockdown (data not shown). 5 embryos loaded per lane. Positive control for Sec10 was from human Sec10 (hSec10) overexpressing MDCK cell lysates. Blot was probed with antibodies against hSec10, and gamma-tubulin as a loading control. (B-C′) Immunofluorescence with antibody against acetylated-tubulin (green), and the nuclear Hoechst stain (blue). Flattened Z-series from confocal imaging of medial (B,C) and posterior kidney (B′,C′), 24 hours post fertilization (hpf), lateral view, 80x magnification. Pronephric cilia length is similar between uninjected embryos (B/B′) and 15ng sec10MO embryos (C/C′); however, cilia within the medial pronephros are disordered (compare B and C). (D-E′) JB-4 resin section (with enlarged inset) of glomerular region, stained with Hematoxylin and Eosin, 3 dpf, transverse 4 μm section, 40x magnification. Wild-type embryos show an organized U-shaped glomerulus (D/D′), while 15ng sec10MO embryos show disorganization (E/E′).

Two splice-site morpholinos (MOs) against zebrafish *sec10* were designed and injected either alone, 15 ng sec10e2i2-MO1, or as a combined dose of 8 ng sec10e2i2-MO1 +8 ng sec10e3i3-MO2. Hereafter, we will use the following shorthand for such morpholino-injected embryos: “15ng sec10MO embryos” and “8+8ng sec10MO embryos”, respectively. Aberrant splicing was verified by sequencing transcripts from 24 hours post fertilization (hpf) cDNA libraries made from sec10MO embryos. Multiple splicing variants were not observed. Sequencing of the transcript from 15ng sec10MO embryos revealed a 25 bp deletion in exon 2. The same 25 bp deletion and an additional 27 bp deletion in exons 3 and 4 were observed in the 8+8ng sec10MO injected embryos. Both cases result in a truncated 33 amino acid protein product. Furthermore, the level of Sec10 knockdown was assayed directly by Western blot with antibody against human Sec10 (hSec10) [39] (Figure 3A). While some zfSec10 remained at 1 day post fertilization (dpf) in sec10MO embryos, levels were significantly reduced soon thereafter. Therefore, these sec10 splice-site morpholinos can be used to effectively knockdown Sec10.

**Table 1:** sec10MO embryos show variable gross phenotypes at 3 dpf.

	Trial	% Wild-type	n =
Uninjected	1	100%	49
	2	100%	39
	3	98%	111
	4	100%	42
15ng sec10e2i2-MO1	1	73%	74
	2	25%	28
	3	45%	113
	4	24%	97
8ng sec10e2i2-MO1 +8ng sec10e3i3-MO2	1	53%	62
	2	52%	48
	3	64%	137
	4	28%	32
Uninjected	total	99%	241
15ng sec10e2i2-MO1		43%	312
8ng sec10e2i2-MO1 +8ng sec10e3i3-MO2		55%	279

Abnormal gross phenotypes, scored at 3 dpf, included any of the following: smaller body size, small eyes, edema, and curly tail down and curly tail up. Lysates isolated from normal- and abnormal-appearing 3 dpf sec10MO embryos showed similar levels of zfSec10 knockdown (data not shown). The following Fisher's exact tests were performed on the proportions of wild-type embryos: First, examination of the total percentages of wild-type phenotypes in all three conditions showed that the frequency of wild-type embryos differed across conditions (p<10^-15^). Second, examination of the 15ng sec10MO embryos compared to the 8+8ng sec10MO embryos showed that the latter yielded a significantly higher frequency of wild-type embryos (p<0.01).

Given the ciliogenesis defects observed with Sec10 knockdown *in vitro* [39], we predicted that pronephric cilia would be shorter in sec10MO embryos. Surprisingly, pronephric cilia length at 1 dpf appeared normal by immunofluorescence (Figure 3B-3C′). We then assayed whether cilia motility was affected. Whereas mammalian kidney cilia are non-motile, pronephric cilia in the zebrafish are motile [18], [25]. We assayed cilia motility at 2 dpf and, surprisingly, found that it was intact in sec10MO embryos (Videos S1 and S2).

The discrepancy in ciliogenesis phenotypes between Sec10 knockdown *in vitro* and *in vivo* may be explained by incomplete knockdown of Sec10 protein in zebrafish. Since we utilized a splice-site morpholino, it is likely that maternally-deposited RNA and/or protein was sufficient to allow for establishment of the cilia at 1 dpf. Indeed, our Western blot analysis detected Sec10 protein at this time (Figure 3A). Therefore, pronephric cilia may only require Sec10 for initial ciliogenesis, but not for later maintenance of the structure. This may also be true for cilia motility.

Unexpectedly, sec10MO embryos showed defects in pronephric development despite the absence of pronephric cilia structure and motility defects. At 1 dpf, the cilia were disordered specifically within the medial pronephros (uninjected: n = 0/3 disorganized; 15ng sec10MO: n = 1/3 disorganized; 8+8ng sec10MO: n = 3/5 disorganized, compare Figure 3B and 3C), where we have observed dilations and pronephric cysts in other zebrafish cilia mutants [20], [25]. Since the disorganization suggested pronephric tubule dilation, we performed histological analysis to look directly for pronephric defects. At 3 dpf, sec10MO embryos did not show obvious dilations in the pronephros; however, the morphology of the glomerulus was abnormal. Instead of a normal compact U-shaped glomerulus, sec10MO embryos showed disorganization, which may be due to increased cell number (uninjected: n = 0/1 disorganized; 15ng sec10MO: n = 3/5 disorganized; 8+8ng sec10MO: n = 1/2 disorganized, compare Figure 3D′ and 3E′).

Therefore, while *in vivo* Sec10 knockdown did not affect pronephric cilia structure or motility, we still observed defects in pronephric development.

### Knockdown of *sec10* partially phenocopies loss of *pkd2*

Though *sec10* knockdown did not affect ciliogenesis *in vivo*, *sec10* knockdown may still perturb other aspects of cilia function, even if cilia structure and motility are intact. Consistent with this possibility, sec10MO embryos displayed a range of gross phenotypes that have been observed in other zebrafish cilia mutants, including smaller body size, small eyes, edema, and curly tail up [18]-[21], [25], [26], [31]. These phenotypes were variably penetrant (Table 1), despite similar levels of protein knockdown (Figure 3A, data not shown). These phenotypes suggest that while a residual level of maternal Sec10 protein was adequate to maintain cilia structure in sec10MO embryos, higher levels are required for full wild-type cilia function.

**Figure 4:**
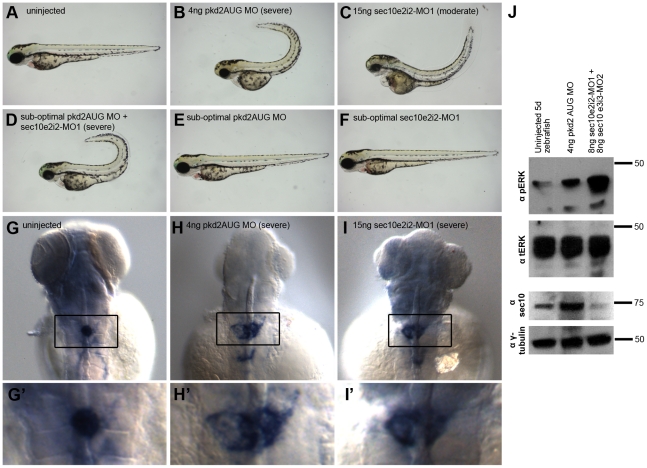
Knockdown of *sec10* partially phenocopies loss of *pkd2*. (A-F) Gross phenotypes of zebrafish embryos at 3 dpf, lateral view, 4x magnification. Uninjected embryo (A), 4ng pkd2MO embryo with a severe curly tail up (B), 15ng sec10MO embryo with a moderate curly tail up (C). A synergistic interaction resulting in severe curly tail up was observed upon co-injection of sub-optimal doses of 0.25/2ng pkd2 MO +7.5ng sec10MO (D)—which do not result in curly tail up when injected alone (E, F). (G-I′) *in situ* hybridization for *wt1a* (with enlarged insets), 3 dpf, dorsal view, 16x magnification. An uninjected embryo with condensed glomerular stain (G/G′), a 4ng pkd2MO embryo with severe enlargement (H/H′), and a 15ng sec10MO embryo with severe enlargement (I/I′). (J) Increased phospho-ERK levels detected by Western blot in 4ng pkd2MO and 8+8ng sec10MO embryos at 5 dpf. One blot, loaded at 2 embryos per lane, was probed with antibody against phospho-ERK, then with antibody against total-ERK. The other blot, with the same lysates as above loaded at 10 embryos per lane, was probed with antibody against hSec10, then with antibody against gamma-tubulin.

One way in which *sec10* could be important for cilia function is through regulating *pkd2* function. Notably, *pkd2* knockdown in zebrafish does not produce defects in cilia structure [29]-[31] or motility [25], [30]—similar to what we observed with Sec10 knockdown by splice-site morpholinos. Since *pkd2* is specifically known to be important for cilia function, and our *in vitro* analysis revealed ADPKD-like behaviour in Sec10 knockdown cells, we wanted to determine whether Sec10 knockdown would share phenotypes associated with *pkd2* knockdown *in vivo* as well. Importantly, we noticed that the curly tail up phenotype of sec10MO embryos was reminiscent of the unique curly tail up observed from loss of *pkd2* in zebrafish (uninjected: 0% curly tail up, n = 42; compared to 15ng sec10MO: 51%, n = 97; and 8+8ng sec10MO: 6%, n = 32; Figure 4A and 4C) [20], [29]-[31].

To determine whether sec10MO embryos shared other phenotypes with pkd2MO embryos, we investigated whether sec10MO embryos displayed left-right defects. Indeed, like pkd2MO embryos, sec10MO embryos show defects in left-right patterning with respect to the positioning of the visceral organs (Table 2). Additionally, we observed defects in asymmetric *nodal* gene expression in sec10MO embryos (Table 2), as would be expected if cilia function was disrupted. Ciliary function in Kupffer's vesicle is known to be upstream of asymmetric *nodal* expression [28]. Thus, loss of *sec10* may affect left-right patterning indirectly through its effects on cilia and/or polycystin-2 function.

**Table 2:** Left-right defects in sec10MO embryos.

	Asymmetric gene expression 19ss				
	Left	Right	Bilateral	n =	*p*
Uninjected	98%	0%	2%	45	—
4ng pkd2AUG MO	30%	7%	63%	46	<10^-6^
15ng sec10e2i2-MO1	66%	17%	17%	48	<0.01
8ng sec10e2i2-MO1 +8ng sec10e3i3-MO2	73%	5%	22%	37	<10^-5^

	Laterality of organ placement 2 dpf				
	Situs solitus	Situs inversus	Heterotaxia	n =	
Uninjected	94%	2%	4%	94	—
4ng pkd2AUG MO	66%	22%	12%	50	0.06
15ng sec10e2i2-MO1	77%	15%	8%	65	<0.01
8ng sec10e2i2-MO1 +8ng sec10e3i3-MO2	73%	5%	22%	58	<10^-3^

Left-right defects assayed at the level of asymmetric gene expression and organ placement as described [31]. Embryos were scored by *in situ* hybridization for asymmetric gene expression of *spaw* (in the lateral plate mesoderm), *lefty2* (in the heart), and *lefty1* (in the diencephalon) by *in situ* hybridization at approximately 19 somite stage (19ss). Embryos were scored for the laterality of organ placement using *myl7* (in the heart), *foxa3* (in the liver), and *insulin* (in the pancreas) at 2 dpf. *Situs solitus* = right-looped heart, left-sided liver, right-sided pancreas. *Situs inversus* = left-looped heart, right-sided liver, left-sided pancreas. Heterotaxia = all other organ conformations. *P* values reported are from Fisher's exact test, comparing the given row against uninjected embryos. We also employed the same test to compare the effects of the various treatments. All treatments differed from one another in their effects on both gene expression and organ placement (*p* = 0.003–2.08x10^-7^) except for the effects of 4ng pkd2MO and 8+8ng sec10MO on organ placement, which were statistically indistinguishable (*p* = 0.19).

Also similar to pkd2MO embryos [29], sec10MO embryos showed glomerular expansion in the pronephros by *in situ* hybridization with the glomerular marker, *Wilm's tumor 1a* (*wt1a)* at 3 dpf (Figure 4G-4I′). Wild-type embryos showed a condensed glomerular stain (100% condensed, n = 30). By contrast, only 50% of 15ng sec10MO embryos showed a condensed stain (50% condensed, 31% moderate enlargement, 19% severe enlargement, n = 16), similar to 4ng pkd2MO embryos (35% condensed, 59% moderate enlargement, 6% severe enlargement, n = 32). Given the glomerular disorganization we observed by histology (Figure 3E′), the expansion in the *wt1a* stain in sec10MO embryos may be due to increased cell proliferation.

In spite of the expanded glomerulus, sec10MO embryos did not display obvious glomerular dilations by histology, nor did they show pronephric cysts. While we note that the lack of dilation in sec10MO embryos contrasts with the glomerular dilation observed in pkd2MO embryos [20], [25], [30], zygotic *pkd2* mutants do not show glomerular dilation either [20], [31]. It is likely that maternal contribution of polycystin-2 and Sec10 explains why we do not observe glomerular dilation or pronephric cysts in *pkd2* mutants and sec10MO embryos, respectively.

To determine whether these *in vivo* pronephric defects were accompanied by the cellular phenotypes we observed with Sec10 knockdown *in vitro*, we assayed pERK levels by Western blot to determine the level of MAPK activation. Consistent with our *in vitro* results, pkd2MO and sec10MO embryos showed abnormally increased pERK levels at 5 dpf, relative to uninjected embryos, at 5 dpf, (Figure 4J). MAPK activation was more pronounced in sec10MO than in pkd2MO embryos.

Therefore, sec10MO embryos share curly tail up, left-right, pronephric, and cellular phenotypes with pkd2MO embryos. Consistent with our *in vitro* analysis, we observed that loss of *sec10* partially phenocopies loss of *pkd2*, supporting the idea that exocyst function is required for polycystin-2 function. Furthermore, our observation of these cilia-related phenotypes is consistent with a role for *sec10* in cilia function, even though overt defects in cilia length and motility were not observed upon knockdown.

### *sec10* and *pkd2* genetically interact for cilia-related phenotypes

Our *in vitro* and *in vivo* analyses together support a link between exocyst *sec10* and the ADPKD gene *pkd2*. While the shared curly tail up phenotype is more specific to *pkd2*, the left-right defects and *wt1a* expansion phenotypes shared between sec10MO and pkd2MO embryos have also been observed upon knockdown of other ciliary proteins [18], [24]. We therefore wanted to directly test for a specific genetic interaction between these two genes. We titrated both sec10 and pkd2 morpholinos to find suboptimal doses that did not result in strong gross phenotypes on their own. Interestingly, when we co-injected both morpholinos at these reduced doses we observed a striking synergistic effect on the curly tail up phenotype (Figure 4D-4F, Figure S1A, S1A′). Co-injection of 0.25ng pkd2MO and 7.5ng sec10MO yielded curly tail up phenotypes when each morpholino alone produced completely wild-type tails. Likewise, co-injection with a slightly higher dose of pkd2MO (2ng pkd2MO) shifted almost all embryos from a range of curly up phenotypes into a severe curly up phenotype. We also observed effects upon the left-right defect (Figure S1B and S1B′) and the *wt1a* glomerular expansion (Figure S1C and S1C′) phenotypes. We had to use different suboptimal doses of pkd2 morpholino for the phenotypes because left-right defects, curly tail, and pronephric phenotypes are extremely dose-sensitive to *pkd2* levels [31]. The genetic interaction we observed between *sec10* and *pkd2* morpholinos suggests that *sec10* may play a role in *pkd2* function in multiple cilia-related processes.

The observed genetic interaction can be interpreted in two ways: *pkd2* and *sec10* may act in parallel pathways, or in the same pathway. In the former case, redundancy between two parallel pathways—one requiring *pkd2* and one requiring *sec10*—explains the lack of phenotypes in the single suboptimal dose conditions; a slight reduction of both pathways upon co-injection of both morpholinos leads to a failure to complement. In the latter case, where *pkd2* and *sec10* act in the same pathway, reduction of function at two steps prevents wild-type function in a dosage-sensitive manner. This latter interpretation is supported by a similar synergistic effect that was observed between the two ciliary proteins Seahorse and Inversin [23]; these proteins were then shown to biochemically interact, supporting the idea that they are likely to act in the same pathway [23], [24]. Previously, we proposed that Sec10 and the exocyst are important for transporting proteins important for ciliary structure [39]. We, therefore, suggest a similar model for transporting proteins important for ciliary function, like polycystin-2. If Sec10 is similarly required to transport polycystin-2, then morpholino co-injection would further impair ciliary polycystin-2 levels beyond that seen following direct knockdown of polycystin-2 by a sub-optimal dose of morpholino, because a reduced amount of Sec10 would be present to effectively transport the remaining polycystin-2.

### The exocyst and polycystin-2 interact biochemically and co-localize in the primary cilium

**Figure 5:**
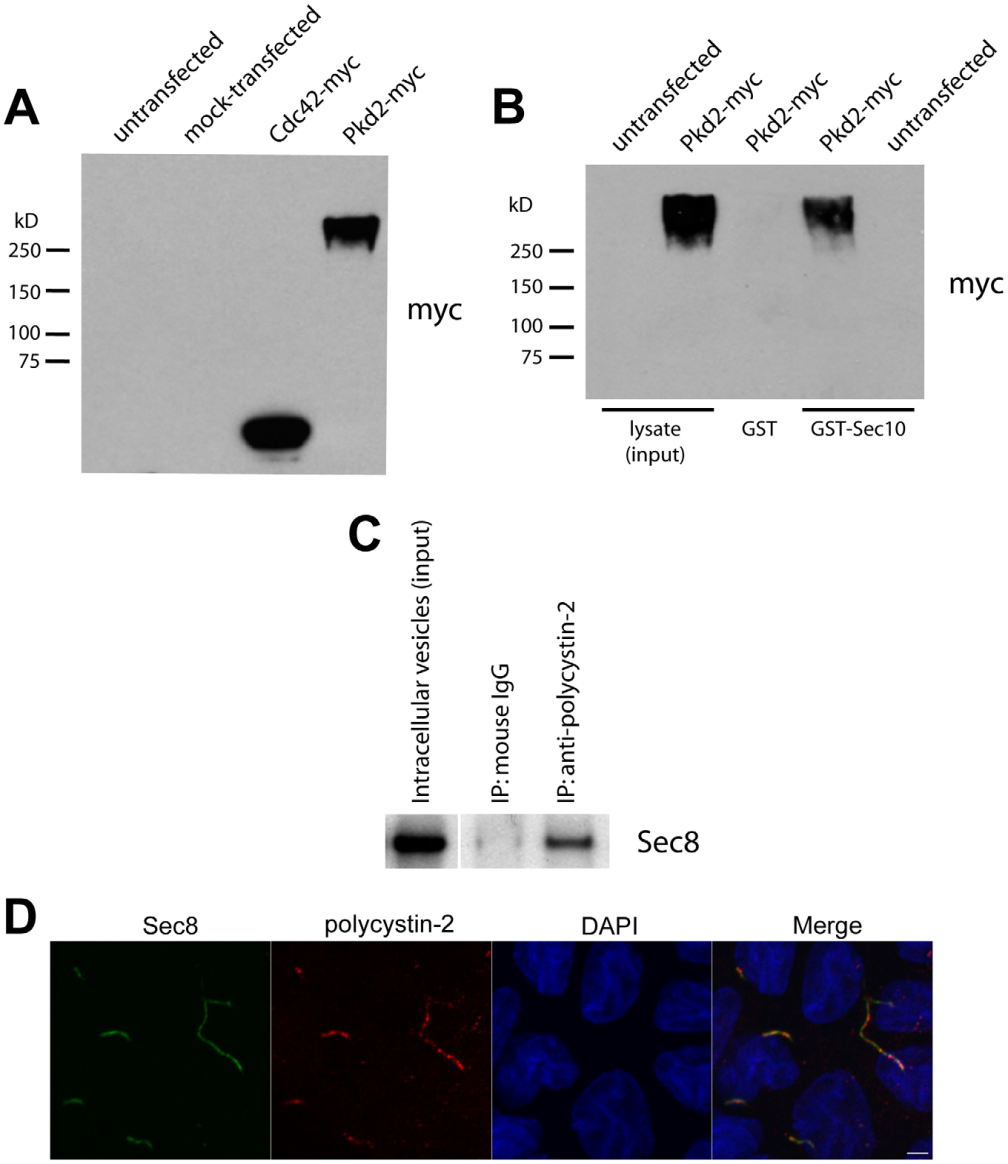
Sec10 biochemically interacts with polycystin-2. (A) Human PKD2-myc in pcDNA3 was transfected using lipofectamine into HEK293 cells. Western blot was performed on the lysate (labeled “Pkd2-myc”), using antibody against the myc epitope tag. Polycystin-2-myc is seen, but at a higher molecular weight than expected, suggesting that the polycystin-2-myc is in a polymeric form. Identical results were seen using a monoclonal antibody against polycystin-2 (data not shown). “Cdc42-myc” = lysate from MDCK cells expressing Cdc42-myc (a positive control for the Western blot). (B) Purified Sec10-GST, but not GST alone, pulled down polycystin-2-myc from PKD2-myc transfected HEK293 cell lysate. (C) Exocyst Sec8 co-immunoprecipitated with polycystin-2, but not the isotype control, from intracellular vesicles isolated from mouse kidney lysate. The lanes in (C) were all from the same gel, though the intracellular vesicle input lane was separated from the other lanes (denoted by a white line). (D) Immunofluorescence staining, using a monoclonal antibody against exocyst Sec8 (green) and a polyclonal antibody against polycystin-2 (red), demonstrated co-localization of endogenous exocyst and polycystin-2 at the primary cilium (yellow in the merged panel) in MDCK cells grown on a Transwell filter for ten days. The panel showing DAPI-stained cell nuclei (blue) was taken at a different level inside the cell than the panels for Sec8 and polycystin-2, and is included here to delineate individual cells. Bar = 1 μm.

In light of the genetic interaction we observed between *sec10* and *pkd2*, we wanted to determine whether we could detect a biochemical interaction between Sec10 and polycystin-2. Using lipofectamine, we transfected a cDNA encoding human polycystin-2-myc into human embryonic kidney 293 (HEK293) cells. Western blotting of the transfected HEK293 cell lysates with antibodies against both polycystin-2 (not shown) and the myc epitope tag (Figure 5A), identified a band at approximately twice the molecular weight of polycystin-2, suggesting it was in polymeric form. Purified Sepharose-immobilized Sec10-GST was then used as an affinity resin to pull down specific binding proteins from polycystin-2-myc transfected HEK293 cell lysates. Western blotting, using antibodies directed against both polycystin-2 and the myc epitope tag, identified polycystin-2 as a Sec10-GST binding protein that was not recovered on the GST resin alone (Figure 5B). Using another technique, we showed that exocyst Sec8 co-immunoprecipitated with polycystin-2, but not the isotype control, from intracellular vesicles isolated from mouse kidney lysate (Figure 5C). Having shown that the exocyst and polycystin-2 can interact in cell lysates, we next investigated if native polycystin-2 would co-localize with the exocyst at the primary cilium. We previously showed by immunofluorescence and electron gold microscopy that the exocyst localized to the primary cilium in MDCK cells [39]. Using a polyclonal polycystin-2 antibody that recognizes canine polycystin-2 [55], we first demonstrated that polycystin-2 co-localizes with acetylated alpha tubulin at the primary cilium in MDCK cells (Figure S2). We then showed that exocyst Sec8 co-localizes along the length of the primary cilium with polycystin-2 (Figure 5D).

Consistent with our phenotypic and genetic analyses in zebrafish, we observe that Sec10 and polycystin-2 biochemically interact and co-localize in the cilia of cultured renal tubule epithelial cells.

### Sec10 biochemically interacts with the ciliogenesis proteins IFT88 and IFT20

Since we observed a biochemical interaction between the exocyst and polycystin 2, we wanted to determine whether the exocyst biochemically interacts with other ciliary proteins. We focused on ciliary proteins such as IFT88 and IFT20, because they have both been found to interact with polycystin-2 [56], [57].

**Figure 6:**
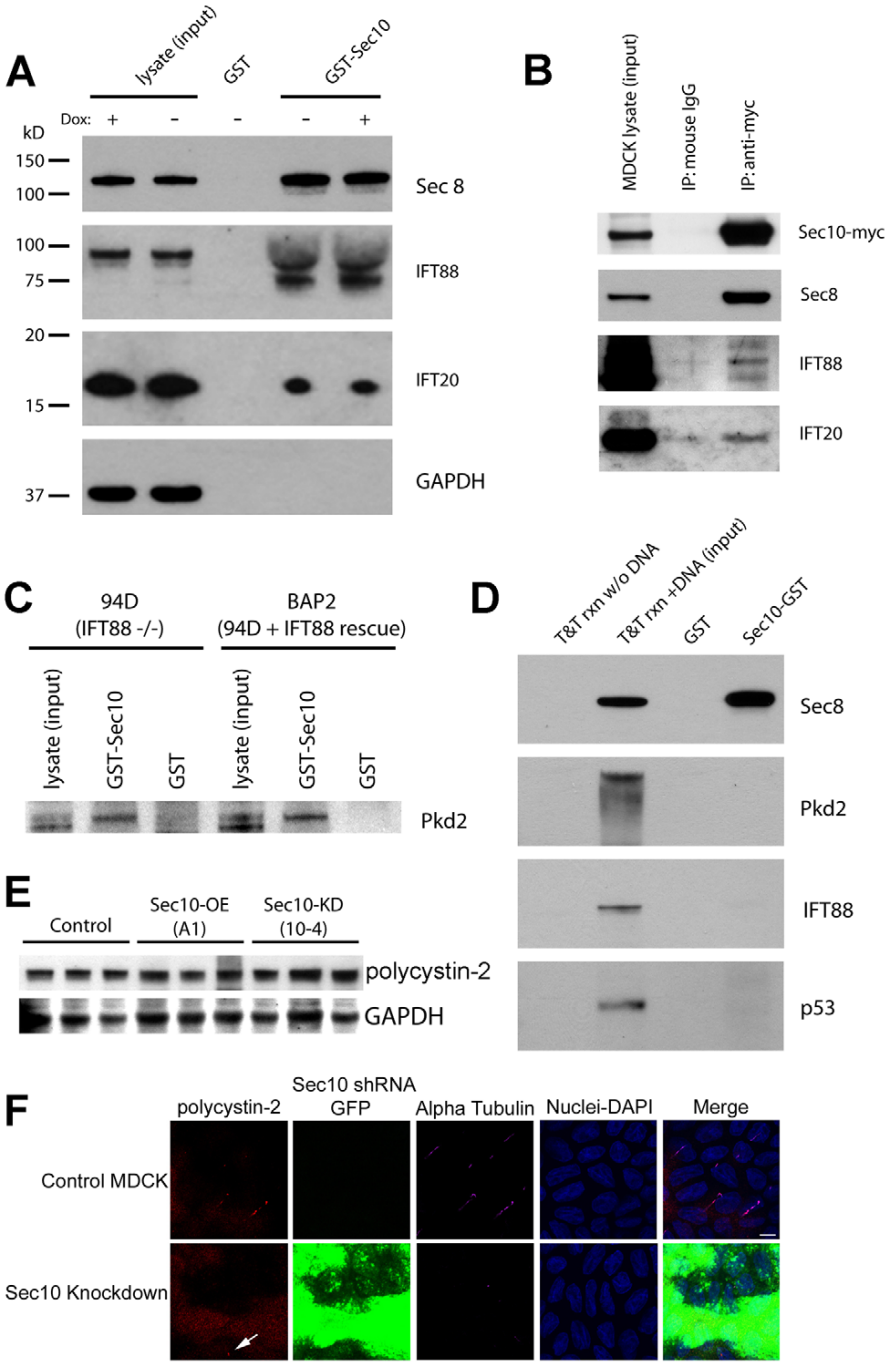
Sec10 interacts with IFT88 and IFT20. (A) After incubation with HEK293 cell lysate, Sec10-GST, but not GST alone, pulled down the cilia transport proteins IFT88 and IFT20. As a positive control for exocyst binding, Sec8 is shown to bind to Sec10-GST. To demonstrate specificity for the pulldown products, we blotted for GAPDH, a house-keeping protein not known to interact with the exocyst. GAPDH was identified only in cell lysate, and not in pulldown fractions. (B) IFT88 and IFT20 co-immunoprecipitated with Sec10, but not isotype controls, from lysates of ciliated MDCK cells expressing a myc epitope-tagged Sec10. (C) To determine if IFT88 was necessary for the interaction between polycystin-2 and Sec10, Sec10-GST pulldowns were performed using lysate from both IFT88-deficient (94D), and IFT88-replete (BAP2) cell lines. There was no difference in the amount of polycystin-2 pulled down from the IFT-deficient or -replete cell lines. (D) *In vitro* translation of Sec8, polycystin-2, IFT88, and p53, followed by Sec10-GST pulldown, was also performed and an interaction was only detected between Sec8 and Sec10. This suggests that the Sec10/polycystin-2 and Sec10/IFT88 interactions are indirect. (E) Equal amounts of polycystin-2 protein, as determined by Western blot, are seen in control (T23), Sec10-overexpressing (A1), and Sec10 knockdown (10-4) MDCK cells. GAPDH staining is presented as a loading control. (F) Immunofluorescence staining, using a monoclonal antibody against acetylated alpha tubulin, that is specific for primary cilia (purple), and a polyclonal antibody against polycystin-2 (red), demonstrated co-localization of polycystin-2 at the primary cilia in control cells. As we previously reported [39], no, or few, primary cilia were seen in Sec10 knockdown cells, and the polycystin-2 appeared to be widely dispersed inside these cells. We occasionally saw concentrated polycystin-2 at what appeared to be the basal body or primary cilium (arrow); however, this occurred in cells demonstrating lower or no GFP expression, indicating a lack of Sec10 knockdown in those cells. The images presented are all X-Y sections, and were obtained using the same confocal settings. Please note that the DAPI staining for the cell nuclei was captured at a different level in the cell than the other sections and is included in the merge to delineate cell boundaries. Bar = 5 μm.

Previously we demonstrated that levels of IFT88 were reduced upon Sec10 knockdown *in vitro* [39]. IFT88 is required for cilia structure [58] and is mutated in the *orpk* mouse model of PKD [59]. Interestingly, IFT88 has been shown to be in a complex with polycystin-2 and possibly trafficked together with it in the cell [57]. Consistent with this interpretation, IFT88 is not itself required for polycystin-2 localization to primary cilia in cultured cells [56]. We used GST-pulldown assays to test for biochemical interactions, as relatively larger amounts of binding proteins can be obtained from the affinity column. A Sec10-GST fusion protein was purified on glutathione Sepharose and used as an affinity matrix for the purification of specific binding proteins from HEK293 cell lysates. IFT88 interacted with Sec10 and was found in the pulldown fraction (Figure 6A). To demonstrate specificity, we probed for GAPDH, a protein not known to interact with the exocyst. In Figure 6A (bottom), GAPDH is seen in the cell lysate, but not in the Sec10-GST pulldown fractions. The glutathione-Sepharose immobilized Sec10 also pulled down exocyst Sec8 from the cell lysate, serving as a positive control for exocyst binding (Figure 6A).

IFT20 is a ciliary protein known to be trafficked to cilia on vesicles, and knockout of IFT20 leads to polycystic kidney disease in mice [56], [60]. Interestingly, IFT20 has been shown to be important for polycystin-2 localization to the primary cilium [56]. IFT20 was also found in the pulldown fraction, and is, therefore, a Sec10 binding partner (Figure 6A). As a negative control, in all the pulldown experiments bead-immobilized GST alone was used and no proteins were detected in the pulldown fractions.

Using another technique, we showed that Sec8, IFT88, and IFT20 all co-immunoprecipitated with Sec10-myc from MDCK cell lysates, but not the isotype control (Figure 6B). Therefore, Sec10 biochemically interacts not only with proteins important in cilia function (like polycystin-2), but also with proteins implicated in cilia formation and trafficking, such as IFT88 and IFT20.

### IFT88 is not required for the biochemical interaction between Sec10 and polycystin-2

Given that IFT88 has been shown to interact with polycystin-2 [57], we next investigated if IFT88 might act as a direct bridging protein between Sec10 and polycystin-2. An immortalized cell line, 94D, derived from the cortical collecting duct cells of an Oak Ridge Polycystic Kidney mutant mouse (*orpk*), and deficient in IFT88, was generated by Yoder and colleagues [61]. An immortalized IFT88 “rescue” cell line, BAP2, was also generated with endogenous levels of IFT88. To determine if IFT88 is necessary for the interaction between polycystin-2 and Sec10, Sec10-GST pulldowns using lysate from both the 94D and BAP2 cell lines were performed. There was no difference in the amount of polycystin-2 pulled down from the IFT88-deficient or -replete cell lines (Figure 6C). Therefore, IFT88 is not required for the Sec10 interaction with polycystin-2.

We then performed *in vitro* translation of Sec8, polycystin-2, IFT88, and p53, followed by pulldown with Sec10-GST. We demonstrated an interaction between Sec10 and Sec8 (our positive control), but not p53 (our negative control), polycystin-2, or IFT88 (Figure 6D). Therefore, while the exocyst biochemically interacts with polycystin-2 and IFT88, these interactions are not direct, indicating there are remaining proteins yet to be identified in these complexes.

### Polycystin-2 is mislocalized upon knockdown of *sec10*

As determined by Western blot, equal amounts of polycystin-2 are seen in control, Sec10-overexpressing, and Sec10 knockdown MDCK cells (Figure 6E). If Sec10 is required for polycystin-2 localization, we would expect to see a change in polycystin-2 localization in Sec10 knockdown MDCK cells. Indeed, Sec10 knockdown cells show a loss of native polycystin-2 localization at the primary cilium by immunofluorescence staining (Figure 6F). However, it should be noted that this result is not surprising given that Sec10 knockdown cells, as we previously reported, have no, or few, cilia [39]. Therefore, the loss of polycystin-2 localization could well be an indirect effect of the ciliogenesis defect.

## Discussion

Here, we describe the first genetic and biochemical link between the exocyst and a human disease gene, PKD2. Phenotypic analyses of sec10MO embryos support a role for *sec10* in multiple cilia-related processes, which is surprising given the absence of obvious defects in cilia structure or motility. Thus, while our previous work indicated a role for Sec10 in ciliogenesis [39], the results presented here suggest that Sec10 is important for cilia function as well. Furthermore, we demonstrate a specific genetic interaction with *pkd2*, a gene that influences cilia function without affecting cilia structure. Knockdown of Sec10 *in vitro* and *in vivo* partially phenocopies knockdown of the ADPKD protein polycystin-2. Consistent with these results, we observe that Sec10 and polycystin-2 co-localize in the primary cilium *in vitro*. We further report biochemical interactions between multiple exocyst proteins and ciliary proteins—polycystin-2, IFT88, and IFT20. Together with our previous work [39], our studies demonstrate that the exocyst protein Sec10 is likely to be important for both cilia formation and function.

We demonstrate that Sec10 knockdown also leads to phenotypes associated with ADPKD. *In vitro*, we show that knockdown of Sec10 leads to decreased basal intracellular calcium levels and lack of a calcium response to fluid flow. Conversely, Sec10-overexpressing cells showed a significantly increased calcium response to fluid flow. These results were not unexpected given the defects in ciliogenesis in Sec10 knockdown cells, and the increased ciliogenesis seen in Sec10-overexpressing cells [39]. We also showed increased cell proliferation in Sec10 knockdown cells. Increased cell proliferation is a well-known characteristic of ADPKD cells and plays a major role in the formation of the cysts that destroy the kidney, leading some to refer to ADPKD as “neoplasia in disguise” [47].

Our analysis of the role *sec10* plays in zebrafish development then revealed that Sec10 may also be required for cilia function, separate from a role in cilia formation. When we used morpholinos to knockdown Sec10 levels *in vivo* in zebrafish, we did not observe a gross defect in pronephros cilia morphology or motility. While this was initially surprising because *in vitro* knockdown results in severe ciliogenesis defects [39], we believe that maternal Sec10—which is unaffected by our splice-site morpholinos—is sufficient to allow for cilia assembly. However, we believe this residual maternal protein was unable to restore complete cilia function during zebrafish development because sec10MO embryos still showed cilia-related phenotypes that have been observed in other cilia mutants in zebrafish—such as left-right patterning defects and glomerular expansion. Therefore, we believe the partial knockdown of Sec10 levels *in vivo* with splice-site morpholinos allowed us to uncover a role for exocyst Sec10 in cilia function.

If true, we would expect that future studies knocking down both maternal and zygotic Sec10 with a start-site morpholino might recapitulate a ciliogenesis defect like that observed *in vitro*. We tested one start-site morpholino but it did not effectively knockdown Sec10 (see Materials and Methods). It should also be noted that even translation blocking morpholinos do not always produce maternal and zygotic losses seen in actual maternal zygotic mutants. For example, translation blocking MOs against IFT88 in fish show almost complete loss of protein by Western blot [62], and yet still do not display phenotypes observed in the maternal-zygotic *ift88/oval* zebrafish mutant [63]. Thus, a maternal-zygotic *sec10* mutant would be required to definitively say whether or not Sec10 is required for ciliogenesis in zebrafish, as it is in MDCK cells.

Finally, we provide phenotypic and genetic evidence that *sec10* may be important specifically for *pkd2* function in these cilia-related processes. This is interesting because *pkd2* is one of the causative genes for ADPKD and could explain the ADPKD-like phenotypes we observed upon Sec10 knockdown *in vitro*. Multiple lines of evidence support our interpretation that *sec10* is important for *pkd2* function *in vivo*: 1) *pkd2* knockdown has been implicated in multiple cilia-related processes and is known to affect cilia function, but not structure or motility [14], [29]-[31], [33]; 2) Similar to *pkd2* knockdown, sec10MO embryos share several cilia-related phenotypes—including the curly tail up and MAPK activation phenotypes, even though they similarly do not show defects in cilia length or motility; 3) We observed specific genetic interactions between *sec10* and *pkd2* for multiple cilia-related phenotypes—including curly tail up, left-right defects, and aberrant *wt1a* glomerular expansion.

It will be important to tease apart the extent to which *sec10* phenotypes are explained solely by inhibition of *pkd2* function. We have provided evidence supporting the idea that many of the *sec10* phenotypes are likely due to its regulation of *pkd2*. But it plausible that *sec10* may be important for the function of other ciliary proteins as well. This is supported by the fact that sec10MO embryos possess cilia-related phenotypes, such as small eyes, that are not observed upon loss of *pkd2*.

While we favour a model where *sec10* is required for ciliary function, we recognize that Sec10 may play a different role since some proteins implicated in cilia function also have cilia-independent functions. Many ciliary proteins are not exclusively localized to the cilium, and it has recently been argued that polycystin function in the endoplasmic reticulum is more relevant for the observed curly tail phenotype [64]. Indeed, multiple IFT proteins have recently been shown to have important functions in non-ciliated cells [65].

We observed activation of ERK (MAPK) following knockdown of Sec10 both *in vitro* and *in vivo*. Similar to polycystin-2 knockout in mouse [66], we report that polycystin-2 knockdown in zebrafish results in elevated pERK levels. Since we propose that polycystin-2 function requires the exocyst, it is not surprising that we also observed increased pERK levels upon Sec10 knockdown. It remains to be determined how MAPK activation relates to cystogenesis, since sec10MO embryos did not show pronephric cysts. We did, however, observe glomerular disorganization that could have been the result of unchecked proliferation. So it is possible that while MAPK activity may regulate proliferation associated with cystogenesis, MAPK hyperactivation alone is not sufficient to cause cystogenesis. If true, this may explain conflicting results others have observed using MAPK inhibitors to abrogate cystogenesis. On the one hand, inhibition of ERK activation with the oral MAP/ERK kinase inhibitor, PD184352, largely prevented cystogenesis in the *pcy* mouse model of polycystic kidney disease [50]. On the other hand, inhibition of ERK activation with the MEK inhibitor, U0126, failed to prevent cystogenesis in a Pkd1 mouse model of ADPKD [66]. The effectiveness of these inhibitors may depend on the specific genetic background and the role MAPK activation plays in cystogenesis in that background. While we observed MAPK hyper-activation in both sec10MO and pkd2MO embryos, only pkd2MO embryos show cysts [20], [25], [30].

In light of its known role in trafficking basolateral proteins to the plasma membrane [41]-[44], we propose that the exocyst is required for the delivery of ciliary proteins to the cilium, including polycystin-2. This model would explain why Sec10 knockdown results in defects in both cilia formation and function.

**Figure 7:**
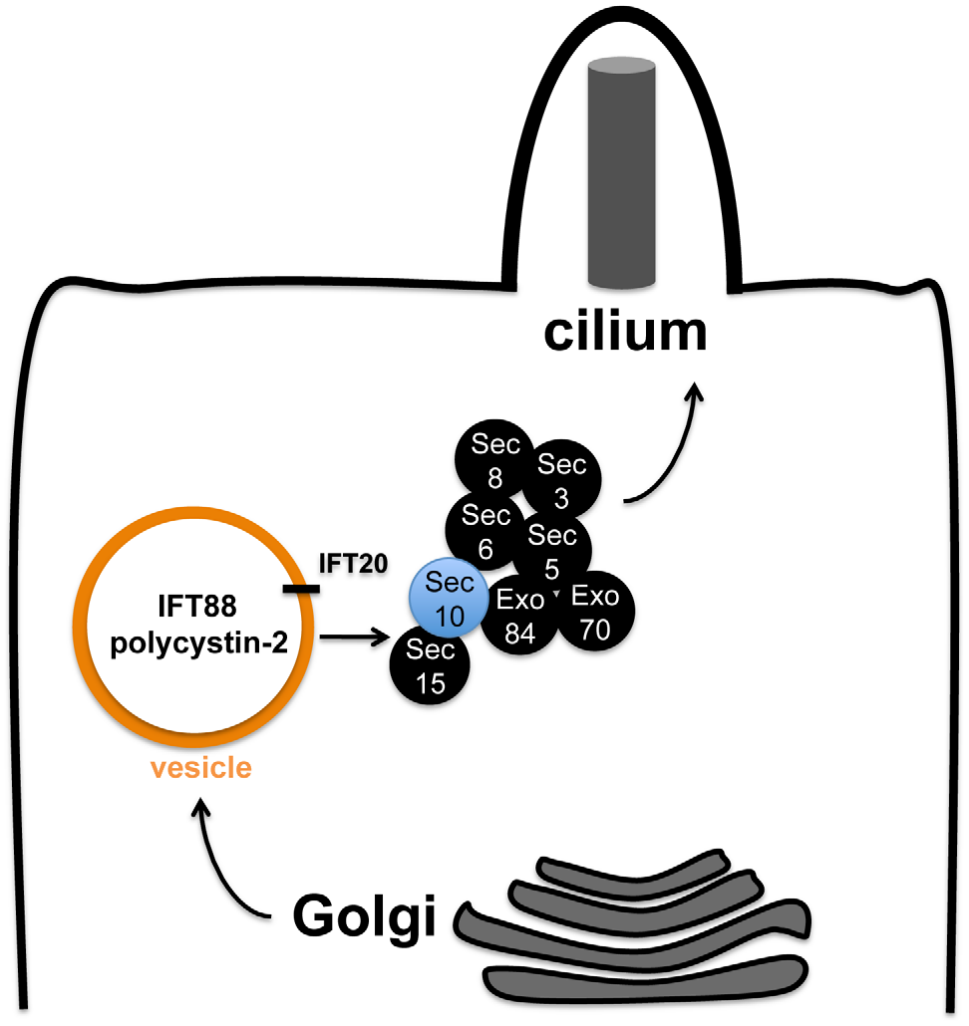
Model for the role of the exocyst in trafficking ciliary proteins. Our model for the trafficking of essential proteins to the primary cilium posits that the exocyst is first localized to the primary cilium, and then targets and docks secretory vesicles from the trans-Golgi network carrying ciliary proteins such as polycystin-2 and IFT88—which are marked by the presence of IFT20.

If our model were true, identification of the IFT B proteins IFT88 and IFT20 in complex with exocyst proteins could mean one of several possibilities: 1) IFT B and polycystin-2 proteins are both being trafficked by the exocyst; 2) The IFT B complex is directly responsible for trafficking polycystin-2 and the exocyst is only directly responsible for trafficking IFT B. We favour the first model (Figure 7) for the following reasons: 1) The biochemical interaction between Sec10 and polycystin-2 that we report did not require IFT88; 2) If the exocyst was required in an indirect fashion for *pkd2* function, through the action of IFT proteins, then it should not have been possible to observe cilia-related phenotypes *in vivo* in the absence of pronephric ciliary structural defects.

Relatedly, IFT20 may play a special role in bridging these complexes since it has been implicated in Golgi-to-cilium vesicular traffic [56]. Unlike other IFT proteins, which are only localized to the cilium, IFT20 is also observed in the Golgi. Pazour and colleagues have proposed that IFT20 may mark vesicles carrying proteins destined for the cilium [56]. IFT20 has been specifically implicated in localizing polycystin-2 to the cilium [56], and polycystin-2 may be trafficked as part of a larger complex containing IFT88 and IFT57 [57]. Our detection of an interaction between Sec10 and IFT20 suggests that IFT20-marked vesicles may utilize the exocyst to dock at the cilium, or that the exocyst may chaperone IFT20-positive vesicles from the Golgi to the primary cilium (Figure 7). Interestingly, IFT20 knockdown by splice-site morpholinos in zebrafish does not significantly disrupt pronephric cilia structure, even though it results in ciliary loss in the otic vesicle [50]. Therefore, loss of IFT20 may result in milder ciliogenesis defects then are observed following knockdown of other IFT proteins in zebrafish.

However, future studies are needed to clarify the interactions among the exocyst, IFT proteins, and the polycystins. Detailed immunofluorescence studies are needed to demonstrate that the exocyst is directly involved in ciliary trafficking. If our model were true, we would expect to see a reduction in the ciliary localization of polycystin-2 upon Sec10 knockdown. Unfortunately, we are unable to test this *in vitro,* which is a limitation of our study, because of the ciliogenesis defects upon Sec10 knockdown in MDCK (Figure 6F) and ARPE-19 cells (data not shown). *In vivo*, we were unable to consistently detect sub-cellular localization of polycystin-2 in the cilium by immunofluorescence utilizing multiple polycystin-2 antibodies (data not shown), thus we were unable to look at polycystin-2 localization in the cilia that remain in sec10MO embryos. Therefore, it remains to be seen how the biochemical interactions we observe between the exocyst and ciliary proteins relate to their trafficking to the cilium.

In summary, we have shown that Sec10, a conserved and crucial component of the exocyst complex, is likely to promote *pkd2* function in many cilia-related phenotypes. These data support an additional role for the exocyst, not only in ciliogenesis [39], but also for cilia function. We report a novel genetic interaction between *sec10* and *pkd2*, which supports the ADPKD-like and *pkd2*-like phenotypes we observed upon Sec10 knockdown *in vitro* and *in vivo*. Our data support a model in which the exocyst is required for the trafficking of proteins essential for ciliary function and structure, possibly in conjunction with IFT20. Future work will reveal the extent to which the exocyst participates in ciliary trafficking, and whether it can be utilized as a novel target for therapeutic intervention in ADPKD, a disease for which there are currently no approved treatments beyond supportive care.

## Materials and Methods

### Ethics statement

All zebrafish experiments were approved by the Institutional Animal Care and Use Committee at Princeton University.

### Cell culture and reagents

All MDCK cell lines used were derived from low passage type II MDCK cells that were obtained from Dr. K. Mostov (UCSF, San Francisco, CA), and which were originally cloned by Dr. D. Louvard (European Molecular Biology Laboratory, Heidelberg, Germany). The monoclonal MDCK type II cell line with silenced expression of Sec10 through stable transfection of shRNA designed against the canine Sec10 gene, as well as the Sec10 overexpression MDCK type II cell line, have been previously described [39]. MDCK cells were cultured on 0.4 µm Transwell filters in modified Eagle's minimal essential medium (MEM) containing Earl's balanced salt solution and L-glutamine, with 5% fetal bovine serum, 100 U/ml penicillin, and 100 µg/ml streptomycin.

The IFT88-deficient and –rescued cell lines, 94D and BAP2, respectively, a generous gift of Dr. Brad Yoder, were derived from the cortical collecting duct cells of the *orpk* mouse, and have been previously described [61]. The orpk mutant mouse has an insertional mutation in the Ift88/Tg737/polaris gene, which results in a hypomorphic allele, with extremely low Ift88 protein levels. This well-studied mutant mouse was crossed with the ImmortoMouse (Charles River Laboratories) and an immortalized cortical collecting duct cell line was established. These cells were transfected with a wild-type Ift88 gene to restore Ift88 expression (BAP2 line), or with an empty vector to retain hypomorphic mutant Ift88 cells (94D line). To keep these cell lines undifferentiated and immortalized, cells were grown at 33°C with 10 U/ml interferon-γ in collecting duct media (DMEM/F-12, 10% FBS, 1.3 µg/l sodium selenite, 1.3 µg/l T3, 5 mg/l insulin, 5 mg/l transferrin, 2.5 mM L-glutamine, 5 µM dexamethasone, 100 U/ml penicillin, and 100 µg/ml streptomycin). To differentiate these cells and inactivate SV40 large-T antigen, cells were grown at 37°C in the absence of interferon-γ for 3 days before performing Sec10-GST pulldowns.

Small molecule inhibitors, which were incubated with cells at the manufacturer recommended concentrations for one hour, include: the MEK inhibitor U0126 at 10 µM (Promega, Madison, WI), PKA inhibitor H89 at 10 µM (Calbiochem, San Diego, CA), Src family inhibitor PP2 at 10 µM (Calbiochem), PKC inhibitor bisindolylmaleimide I (BIM) at 1 µM (Calbiochem), and mTOR inhibitor Rapamycin (Rap) at 20 nM (LC Laboratories, Woburn, MA).

### Cellular assays

Live cell fluorescent imaging and intracellular calcium concentration measurements were done as previously described [46]. Briefly, cells were grown to confluent monolayers on 0.4 µm clear polyester permeable supports in a perfusion chamber, which allows precisely controlled shear fluid flow across the apical surface of the epithelium. After 5-7 days in confluent monolayers, cells were loaded with fura 2 (10 µM fura 2/AM; Teflabs, Austin, TX), and transferred to the incubation chamber on the microscope, where a constant temperature of 37°C was maintained, with a 5% CO_2_ concentration. Cells were then perfused with Ringer's solution with 1 mM probenecid. We performed dual-excitation wavelength fluorescence microscopy (Photon Technologies, Birmingham, NJ) with a Nikon microscope, x20 S Fluor long-working distance objective, and a cooled SenSys charged-coupled camera (Photometrics, Tucson, AZ). Fura 2 was excited at wavelengths of 340 and 380 nm and emitted fluorescence was measured at 510 nm. Data were obtained in each experiment from a grid of 20 regions of interest each containing 8–10 cells. Cells were maintained in the absence of apical flow for 10 minutes followed by an abrupt increase to a flow rate of 5 ml/min. The basolateral flow rate remained constant at 1.5 ml/min.

For cell proliferation measurements, MDCK cells were seeded in parallel 96-well plates in triplicates at 4000 cells/well, and were allowed to attach for 24 h at 37°C. The medium was replenished on all plates, and the cells on one plate were counted using the CellTiter-Glo viability assay (Promega) and considered cell population at t = 0 h. Cells on other plates were grown for 24 h and 48 h at 37°C, and then counted using the same method. Luminescence was measured using a Becton Dickson microplate reader, and proliferation rates were calculated by dividing cell number at a given timepoint by the initial number of seeded cells (t = 0).

### Western analysis

For MDCK cell lysates, immunoblotting was performed as previously described [39]. To make zebrafish protein lysates, embryos were washed in E3 buffer and re-suspended in Ringer's solution. To remove the yolk protein, the sample was vortexed five times in 30 seconds bursts and the supernatant was removed following a gentle centrifugation at 300g for 1 minute at 4°C. The pellet was re-suspended in lysis buffer [67] supplemented with a protease inhibitor cocktail (P1860, Sigma) and 200 µM PMSF. The protein lysate supernatant was removed following centrifugation at 14,000g for 10 minutes at 4°C. Protein samples were mixed with Laemmli buffer and boiled for 5 minutes. Immunoblotting was performed using standard protocols.

The antibodies used in this study are: rabbit polyclonal anti-Sec10 which was described previously [39], rabbit polyclonal anti-phospho-ERK1/2 (#9101, Cell Signaling, Danvers, MA), rabbit polyclonal anti-total ERK1(/2) (sc-94, Santa Cruz Biotechnology, Inc., Santa Cruz, CA), mouse monoclonal anti-GAPDH (G8795, Sigma, St. Louis, MO), rabbit polyclonal anti-γ tubulin (T5192, Sigma), mouse monoclonal anti-acetylated α-tubulin (T6793, Sigma), rabbit polyclonal anti-IFT88 ([68]), mouse monoclonal anti-Sec8 (Assay Designs, Ann Arbor, MI), mouse monoclonal anti-myc (#2276, Cell Signaling), rabbit polyclonal anti-IFT20 ([56], a generous gift from Dr. Greg Pazour), mouse monoclonal anti-polycystin-2 (D-3, Santa Cruz Biotechnology, Inc.), rabbit polyclonal anti-TRPM4 (Santa Cruz Biotechnology), rabbit polyclonal anti-polycystin-2 (a gift from the Johns Hopkins Research and Clinical Core Center), normal isotype mouse IgG control (Santa Cruz), and normal isotype rabbit IgG control (Santa Cruz).

### Zebrafish injections and morpholinos

Embryos were injected at the one-to-8-cell stage, and morpholinos were diluted with phenol red tracer at 5 μg/μL phenol and injected at 500 picoLiter or 1 nanoLiter/embryo. Splice-site morpholinos designed against zebrafish *sec10* were purchased from Gene Tools, LLC. (Philomath, OR): sec10e2i2-MO1 (5′- AATATTCTGTAACTCACTTCTTAGG -3′), sec10e3i3-MO2 (5′-CAAATGTAAAGACGACTGACTTGGT-3′), and sec10AUG-MO1 (5′- CGAATAATTGAGCTGTCGTAGCCAT-3′). Sec10e2i2-MO1 was designed to target the exon 2-intron 2 boundary (hence “e2i2”). Sec10e3i3-MO2 was targeted against the exon 3-intron 3 boundary (hence “e3i3”). Splice-site morpholinos were injected either as a single dose of 15 ng sec10e2i2-MO1 (designated in the text as “15ng sec10MO”) or a combined dose of 8 ng sec10e2i2-MO1 +8 ng sec10e3i3-MO2 (designated in the text as “8+8ng sec10MO”) per embryo. Developmental delay was more noticeable with 8+8ng sec10MO. Sec10e3i3-MO2 did not show gross phenotypes when injected alone. sec10AUG-MO1 was designed to target the ATG (hence “AUG”, complementary sequence underlined above). sec10AUG-MO1 was tested but did not knockdown zfSec10 by Western blot analysis even at doses of 15 ng per embryo (data not shown); since some MOs fail to work for inexplicable reasons, this was not pursued further. pkd2AUG MO (5′-AGGACGAACGCGACTGGAGCTCATC-3′), start site [20], was injected at 4 ng per embryo.

### Immunofluorescence, histology, and *in situ* hybridizations

Immunostaining of MDCK cells grown on Transwell filters was performed as previously described [39], except the cells were fixed with 4% paraformaldehyde for 15 minutes at 37°C. Immunofluorescence for pronephric cilia and histology in zebrafish was performed similar to previously published protocols [25]. The antibodies used in this study for immunofluorescence studies: mouse monoclonal anti-acetylated α-tubulin (T6793, Sigma), rabbit polyclonal anti-polycystin-2 antibody (a gift from the Johns Hopkins Research and Clinical Core Center, [55]), goat-anti-mouse IgG2b/g2b chain specific-FITC (Southern Biotech#1090-02, Birmingham, AL). *In situ* hybridization in zebrafish was performed using standard protocols [69]. Probes used: *wt1a, myl7*, *foxa3*, *ins, spaw*, *lefty1*, and *lefty2*.

### Imaging

All images were captured in TIF format and processed in Adobe Photoshop CS4. For immunofluorescence, zebrafish embryos were imaged on a Zeiss LSM 510 Confocal Microscope with a 40x water objective and captured at 2x zoom, and the LSM Image Browser application; TIFs were captured at 150 ppi. For histology and *in situ* hybridization, samples were imaged using a Leica DM RA2 Microscope, a Leica DFC490 digital camera, and the Leica Application Suite v 3.1.0 software; TIFs were captured at 96 ppi. For live images, embryos were imaged on a Leica MZ FLIII Stereo-Fluorescence Microscope using a Jenoptik LaserOptikSysteme ProgResC14 digital camera and Picture Frame v2.3 software; TIFs were captured at 150 ppi. For video microscopy of pronephric cilia, embryos were imaged as previously described [25], using an Olympus BX51 Upright Microscope with a 60x water immersion objective, an Andor Technology Luca EMCCD digital camera, and Matlab software.

### GST pull-downs and co-immunoprecipitations

Full-length human Sec10 cDNA was cloned in frame into the plasmid pGEX-4T-1 (Amersham Biosciences, Piscataway, NJ), and transformed into the DE3 strain of *Escherichia coli* (Stratagene, La Jolla, CA). GST fusion protein expression was induced by adding isopropyl-1-thio-β-D-galactopyranoside to growing cultures and shaking for an additional 3 h at 37°C. Recombinant proteins were purified with glutathione-Sepharose (Amersham Biosciences) following bacterial cell lysis. For pull-down experiments, lysates from wild-type HEK293 cells, HEK293 cells transfected with PKD2-myc (a generous gift from Dr. S. Solmo), or differentiated 94D and BAP2 cell lines, were incubated overnight with Sec10-GST, or GST only, bound to glutathione-Sepharose. Pull-downs were washed extensively, and then resuspended in Laemmli buffer and boiled, and equal amounts were electrophoresed by SDS-PAGE. Bound IFT88, IFT20, GAPDH, Sec8, and polycystin-2-myc were detected by Western blot analysis.

Co-immunoprecipitations for polycystin-2 were performed from intracellular vesicle fractions of mouse kidney lysates, isolated as described [70] . Isolated vesicles were incubated overnight with polycystin-2 antibody, or equal amounts of control mouse IgG, and protein complexes were precipitated with ProteinG Dynabeads (Invitrogen). After washing five times in vesicle isolation buffer, the precipitated protein complexes were analyzed with SDS-PAGE and Western blotting. Co-immunoprecipitations for Sec10 were performed from confluent MDCK cells overexpressing Sec10 containing a myc epitope tag, since our Sec10 antibody cannot be used for immunoprecipitation. Cells were grown 5-7 days past confluency, washed with PBS, and incubated with 1 mM of the membrane-permeable chemical crosslinker dithiobis(succinimidylpropionate) (DSP) (Thermo Scientific) for 30 minutes at room temperature. The cells were quenched with TBS for 15 min, and then lysed in Co-IP lysis buffer (20 mM HEPES pH 7.4, 120 mM NaCl, 1 mM EDTA, 1% IGEPAL CA-630). Soluble proteins from the lysates were incubated overnight with 2 µg of various antibodies in parallel with equal amounts of isotype control IgG. Protein-G agarose (Invitrogen) was used to precipitate the protein complexes, and after five washes with the Co-IP buffer, the agarose resin was resuspended in Laemmli buffer. Equal amounts of samples were electrophoresis by SDS-PAGE, and co-immunoprecipitated proteins were detected by Western blot analysis utilizing the Trueblot-HRP secondary antibodies (eBioscience).

### Statistical analysis

One-way ANOVAs, with post-hoc Tukey test for statistical significance, were performed to compare band intensities from Western blots and cell proliferation rates using the Prism statistical software (Graphpad, San Diego, CA). Fisher's exact tests for statistical significance were performed to compare phenotypic analyses in zebrafish using R statistical software.

## Supporting Information

Figure S1 *sec10* and *pkd2* genetically interact for cilia-related phenotypes. (A) Genetic interaction for curly tail up phenotype at 3 dpf, using co-injection of 7.5ng sec10MO with either 2ng pkd2MO or 0.25ng pkd2MO. Mild ≤90° tail curve relative to main body axis, Moderate  = 90° curve, Severe ≥90° curve. See Figure 4A-4F for representative images. (B) Genetic interaction for the left-right phenotype of heart jogging at 1 dpf, using co-injection of 7.5ng sec10MO with 0.25ng pkd2MO. (C) Genetic interaction for the *wt1a* glomerular expansion at 3 dpf, using co-injection of 7.5ng sec10MO with 2ng pkd2MO. See Figure 4G-4I′ for representative images of severe phenotype. (A′, B′, C′) *P* values reported are from Fisher exact test results, comparing the conditions designated by the axes. Yellow corresponds to *p*<0.05, Red corresponds to *p*>0.05. If the specific *p* value is not given, an empty yellow box corresponds to *p*<0.001 and an empty red box corresponds to *p* = 1.
(0.42 MB TIF)

Figure S2 Polycystin-2 localizes to the primary cilium in MDCK cells. MDCK cells were grown on a Transwell filter for ten days. Immunofluorescence staining, using a monoclonal antibody against acetylated alpha tubulin (green), which is specific for primary cilia, and a polyclonal antibody against polycystin-2 (red), demonstrated co-localization of native polycystin-2 at the primary cilium in MDCK cells (yellow in the merged panel). The panel showing DAPI-stained cell nuclei (blue) was taken at a different level inside the cell than the panels for acetylated alpha tubulin and polycystin-2, and is included here to delineate individual cells. Bar = 5 μm.
(1.37 MB TIF)

Video S1 Pronephric cilia motility in wild-type embryos. Live video microscopy of pronephric cilia motility in uninjected embryos (n = 1). 2 dpf, lateral view, 60x magnification. Videos were recorded at 100 frames/second, and then processed into AVI movies at 16 frames/second using Matlab software.
(4.01 MB AVI)

Video S2 Pronephric cilia motility is intact in sec10MO embryos. Live video microscopy of pronephric cilia motility in 15ng sec10MO embryos (n = 2). 2 dpf, lateral view, 60x magnification. Videos were recorded at 100 frames/second, and then processed into AVI movies at 16 frames/second using Matlab software.
(4.01 MB AVI)
