# Supplementary figures and images for: The Exocyst Protein Sec10 Interacts with Polycystin-2 and Knockdown Causes PKD-Phenotypes
Source: PLoS Genet. 2011 Apr 7;7(4):e1001361. doi: 10.1371/journal.pgen.1001361 (PMC3072367; doi:10.1371/journal.pgen.1001361)

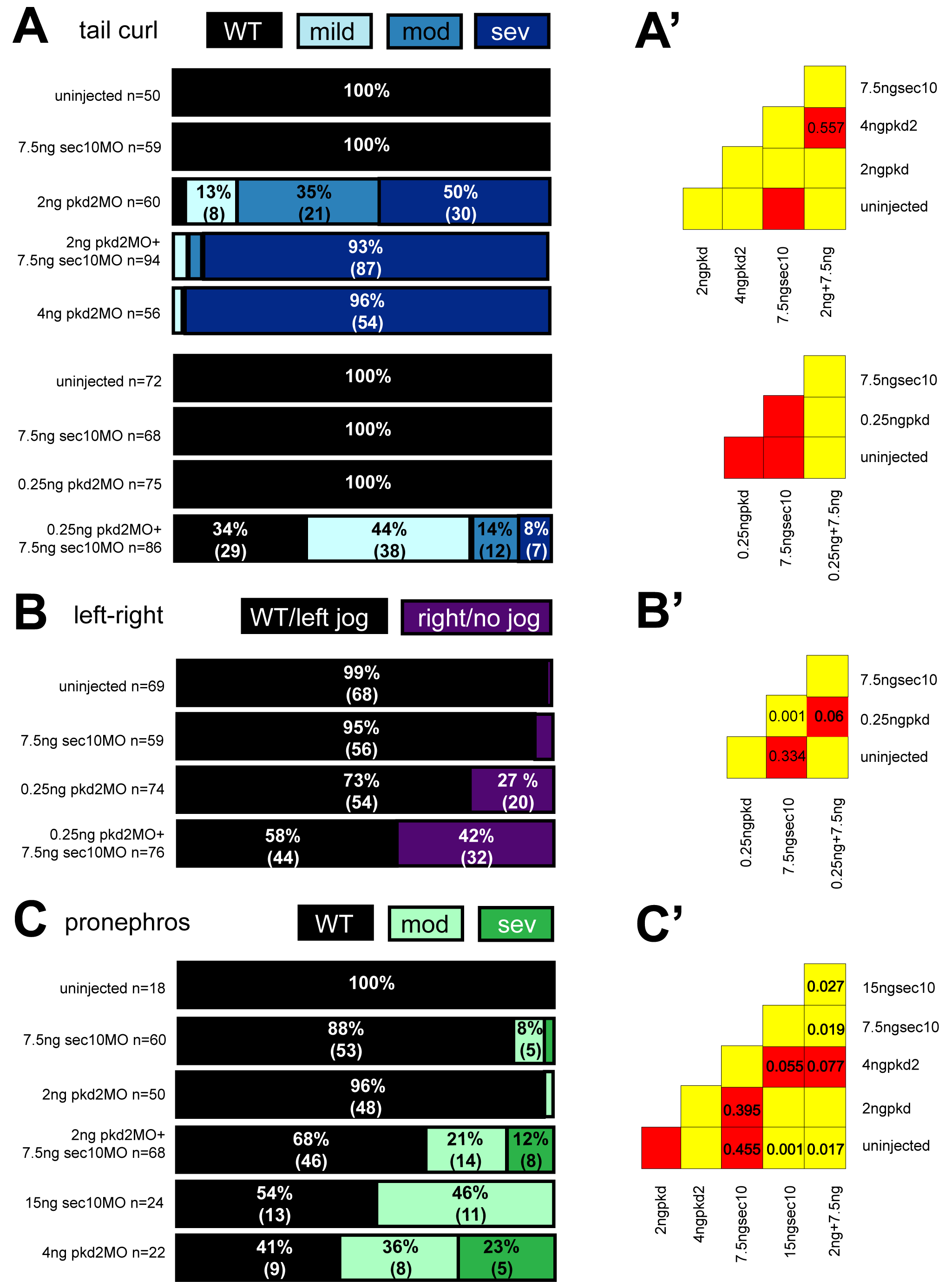

Supplement: Figure S1 — sec10 and pkd2 genetically interact for cilia-related phenotypes. (A) Genetic interaction for curly tail up phenotype at 3 dpf, using co-injection of 7.5ng sec10MO with either 2ng pkd2MO or 0.25ng pkd2MO. Mild ≤90° tail curve relative to main body axis, Moderate = 90° curve, Severe ≥90° curve. See Figure 4A-4F for representative images. (B) Genetic interaction for the left-right phenotype of heart jogging at 1 dpf, using co-injection of 7.5ng sec10MO with 0.25ng pkd2MO. (C) Genetic interaction for the wt1a glomerular expansion at 3 dpf, using co-injection of 7.5ng sec10MO with 2ng pkd2MO. See Figure 4G-4I′ for representative images of severe phenotype. (A′, B′, C′) P values reported are from Fisher exact test results, comparing the conditions designated by the axes. Yellow corresponds to p<0.05, Red corresponds to p>0.05. If the specific p value is not given, an empty yellow box corresponds to p<0.001 and an empty red box corresponds to p = 1. (0.42 MB TIF) [file pgen.1001361.s001.tif]

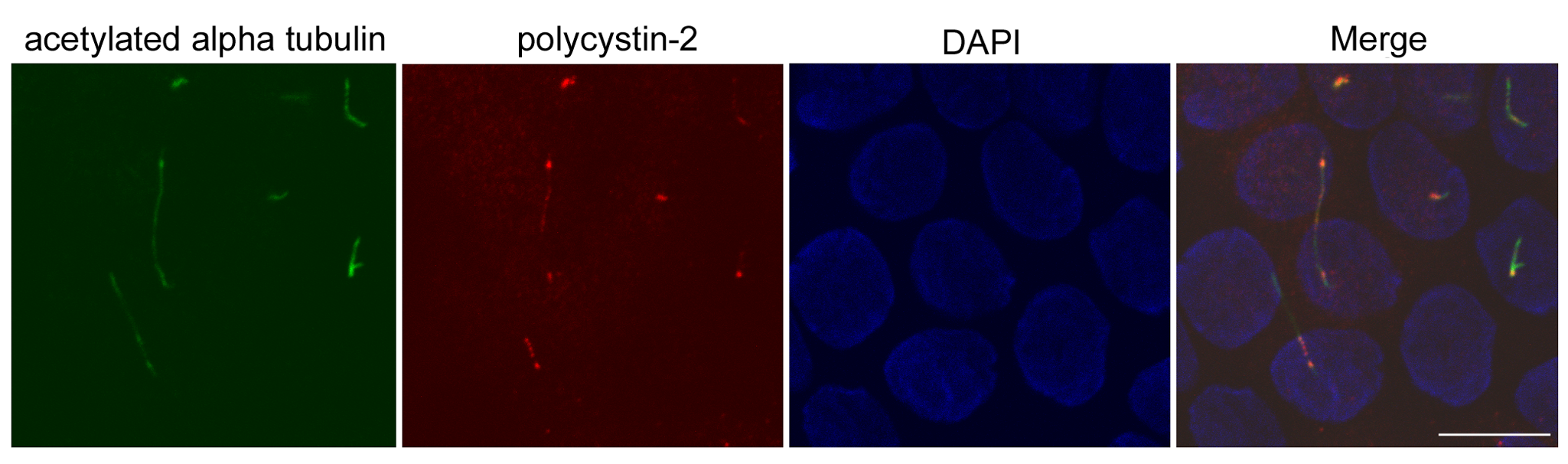

Supplement: Figure S2 — Polycystin-2 localizes to the primary cilium in MDCK cells. MDCK cells were grown on a Transwell filter for ten days. Immunofluorescence staining, using a monoclonal antibody against acetylated alpha tubulin (green), which is specific for primary cilia, and a polyclonal antibody against polycystin-2 (red), demonstrated co-localization of native polycystin-2 at the primary cilium in MDCK cells (yellow in the merged panel). The panel showing DAPI-stained cell nuclei (blue) was taken at a different level inside the cell than the panels for acetylated alpha tubulin and polycystin-2, and is included here to delineate individual cells. Bar = 5 μm. (1.37 MB TIF) [file pgen.1001361.s002.tif]
